# Super‐Durable, Tough Shape‐Memory Polymeric Materials Woven from Interlocking Rigid‐Flexible Chains

**DOI:** 10.1002/advs.202406193

**Published:** 2024-08-05

**Authors:** Jing Xu, Mingchao Shao, Tianze Chen, Song Li, Yaoming Zhang, Zenghui Yang, Nan Zhang, Xinrui Zhang, Qihua Wang, Tingmei Wang

**Affiliations:** ^1^ State Key Laboratory of Solid Lubrication Lanzhou Institute of Chemical Physics Chinese Academy of Sciences Lanzhou 730000 China; ^2^ Center of Materials Science and Optoelectronics Engineering University of Chinese Academy of Sciences Beijing 100049 China; ^3^ Key Laboratory of Science and Technology on Wear and Protection of Materials Lanzhou Institute of Chemical Physics Chinese Academy of Sciences Lanzhou 730000 China

**Keywords:** mechanical properties, molecular weaving, polyurethane, shape memory, supramolecular polymers

## Abstract

Developing advanced engineering polymers that combine high strength and toughness represents not only a necessary path to excellence but also a major technical challenge. Here for the first time a rigid‐flexible interlocking polymer (RFIP) is reported featuring remarkable mechanical properties, consisting of flexible polyurethane (PU) and rigid polyimide (PI) chains cleverly woven together around the copper(I) ions center. By rationally weaving PI, PU chains, and copper(I) ions, RFIP exhibits ultra‐high strength (twice that of unwoven polymers, 91.4 ± 3.3 MPa), toughness (448.0 ± 14.2 MJ m^−3^), fatigue resistance (recoverable after 10 000 cyclic stretches), and shape memory properties. Simulation results and characterization analysis together support the correlation between microstructure and macroscopic features, confirming the greater cohesive energy of the interwoven network and providing insights into strengthening toughening mechanisms. The essence of weaving on the atomic and molecular levels is fused to obtain brilliant and valuable mechanical properties, opening new perspectives in designing robust and stable polymers.

## Graphical abstract

Here for the first time a rigid‐flexible interlocking polymer (RFIP) is reported featuring remarkable mechanical properties, consisting of flexible polyurethane (PU) and rigid polyimide (PI) chains cleverly woven together around the copper(I) ions center. By rationally weaving PI, PU chains, and copper(I) ions, RFIP exhibits ultra‐high strength (91.4 ± 3.3 MPa), toughness (448.0 ± 14.2 MJ m^‐3^), fatigue resistance, and shape memory properties

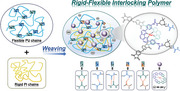

## 1 Introduction

Engineering polymers, with their core competitive mechanical properties (strength, modulus, and toughness), have evolved into indispensable materials for the aerospace, aviation, national defense, and military industries. As treasures in today's materials science, polymers are highly favored by researchers for their overall performance, lightweight processes, and diversified applications, presenting unlimited possibilities for modern engineering and technological innovation.^[^ ^1^, ^2^, ^3^, ^4^, ^5^ ^]^ Superior mechanical properties typically entail higher load‐bearing capacity (strength) at minimal deformation and greater resistance to fracture (toughness) at maximum deformation, an apparent paradox.^[^ ^6^, ^7^, ^8^ ^]^ Consequently, designing advanced polymeric materials that combine high strength, toughness, ductility, and versatility is not only a necessary path to excellence but also a major scientific challenge.

**Scheme 1:**
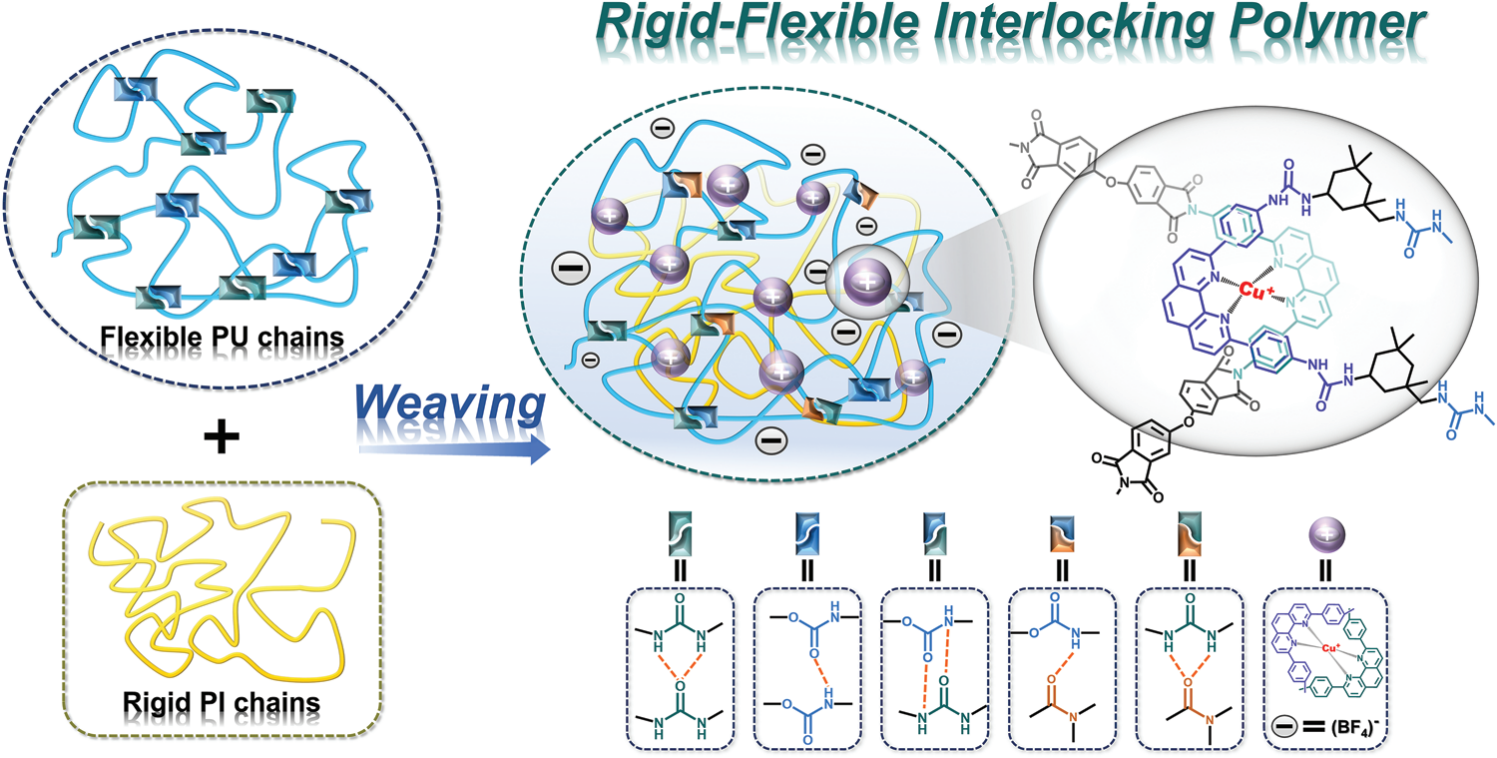
Schematic of weaving PU and PI chains into rigid‐flexible interlocking polymer.

Today, an emerging approach is to regulate the mechanical properties of polymers at the macroscale by exploring the behaviors of their molecular chains at the microscale. The “molecular weaving” introduced by Busch et al.,^[^ ^9^ ^]^ has attracted our great attention. They argued that intertwining patterns between covalent molecular chains can be achieved through periodic orderly entanglements.^[^ ^10^ ^]^ Generally speaking, “weaving” represents the interweaving of long threads and is one of the most durable and efficient ways of creating complicated, ordered materials.^[^ ^11^ ^]^ From its structure, it can be found that macro weaving starts from the nodes and then extends and intertwines in a specific way to form a fabric. Molecular weaving is the orderly entanglement of flexible 1D molecular chains into an extended 2D fabric or 3D network at the molecular level. Recently, Omar Yaghi has woven the first 3D covalent organic framework (COF) in history, named COF‐505, through helical organic “threads”.^[^ ^12^ ^]^ Compared with previous COFs, the cuprous ions in COF‐505 as templates to guide molecular chains to be entangled in a braided manner, showing obvious advantages in structural flexibility, elasticity, and reversibility. Taking inspiration from this, we boldly envisioned the possibility of weaving different macromolecular chains with stable conformations based on non‐covalent bonds to achieve desirable mechanical properties.^[^ ^13^, ^14^ ^]^ Despite some recent advances in molecular weaving, to date, research on weaving macromolecular chains into high‐performance materials is still sparse, especially of the type discussed here.

Herein, we report a RFIP with extraordinary mechanical properties, cleverly woven from two distinct macromolecular chains (rigid and flexible chains). In the molecular network of RFIP, flexible PU chains and rigid PI chains are woven together around the copper(I) ions center (**Scheme** **1**). In other words, the phenanthroline ligands and the π–π interaction between the phenanthroline and neighboring phenyl planes together create a distorted tetrahedral geometry with the Cu(I) ions as templates (registration points) to precisely connect PU and PI chains into a spiral network with woven textures. Results from molecular dynamics (MD) simulations and characterization analysis together supported the correlation between microstructure and macroscopic features, verifying that interwoven molecular chains held greater cohesive energies; therefore, RFIPs exhibited greater strength, toughness, and even modulus compared to non‐woven flexible polymers. On this basis, insights into strengthening toughening mechanisms of RFIPs were presented, involving “hard‐soft” microphase‐separated structures and high energy dissipation conferred by hydrogen bonds (H‐bonds) as well as metal‐ligand coordination bonds formed by Cu(I) ions and phenanthroline ligands. Deriving from these synergistic effects, rigid and flexible polymeric materials are successfully constructed, and their mechanical strength, fatigue resistance, and shape memory behaviors have been well demonstrated.

## 2 Results and Discussion

### 2.1 Molecular Design and Characterizations

**Figure 1:**
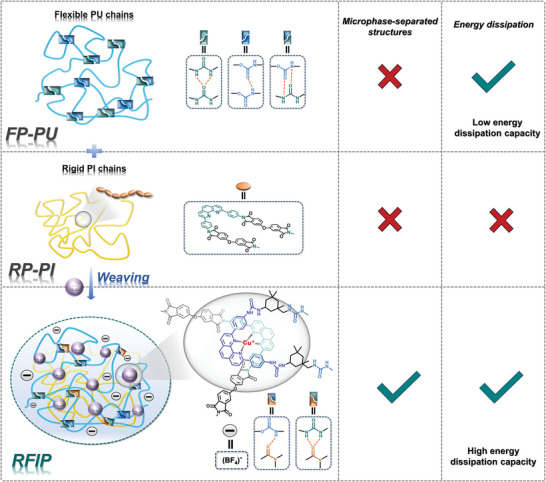
Schematic diagram of weaving PU and PI chains into rigid‐flexible interlocking polymer and strengthening toughening mechanisms.

We heuristically designed a series of rigid‐flexible interlocking polymers (RFIPs) that are dynamically woven from rigid PI and flexible PU chains based on Cu(I) ions, and the process is illustrated in **Figure** **1**. In this study, the flexible PU chain was synthesized from poly(carbamate diol) (PCDL, Mn ≈ 2000), isophorone diisocyanate (IPDI), and two amine chain extenders (4,4′‐diaminodicyclohexyl methane (DDCM) and 4,4′‐(1,10‐phenanthroline‐2,9‐diyl)dianiline (PDA)) via a three‐step reaction process (Figure S1, Supporting Information), and the rigid PI chain was prepared from 4,4′‐oxydiphthalic anhydride (ODA) and PDA via a two‐step process (Figure S2, Supporting Information). Unless otherwise stated, we mainly use strongly phase‐separated PI and PU chains with high modulus contrast between the hard and soft phase networks. The introduced tetrafluoroborate anions occupy the pores and the Cu(I) ions serve as templates to accurately weave the PI and PU chains together in a precise manner. To obtain the best macroscopic mechanical properties through flexible regulation of the microscopic network, we prepared rigid‐flexible polymers with different PI contents, named RFIP‐1, RFIP‐2, and RFIP‐3, respectively. In particular, the ratio of PDA and Cu(I) tetra(acetonitrile) tetrafluoroborate (Cu(MeCN)_4_BF_4_) was always 2:1 to achieve perfect coordination of PDA and Cu(I) ions. As depicted in Figure 1, the long PU chains provide flexible space, while the PI chains contain rigid structures, and the PI chains are entangled on the PU chains, which promotes the generation of “hard‐soft” microphase‐separated structures and imparts high energy dissipation capabilities, thereby obtaining tough shape‐memory polymeric materials, namely RFIPs.

**Figure 2:**
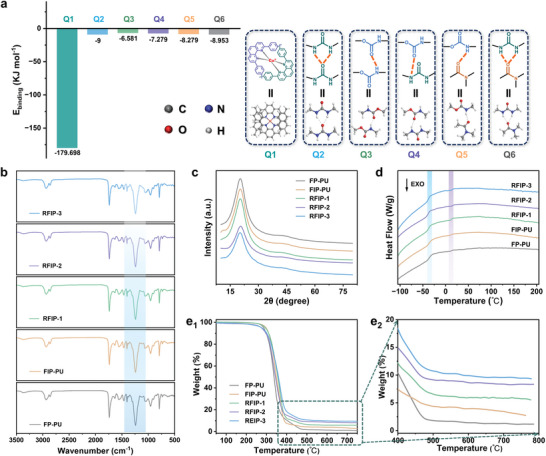
Calculation and general characterization. a) The binding energies of various H‐bonds and metal coordination bonds in the polymers were calculated. b) ATR‐FTIR spectra of all polymers in the range of 3800–500 cm^−1^. c) XRD patterns of all polymers (radiation of wavelength = 0.1541 nm). d) DSC curves of all polymers. e_1_,e_2_) TGA curves of all polymers.

A series of control samples were also prepared: a non‐woven flexible PU polymer (named FP‐PU), a woven flexible interlocking PU polymer (named FIP‐PU), and a non‐woven rigid PI polymer (named RP‐PI). Table S1a,b (Supporting Information) gives detailed synthesis information of all samples, especially noting that RP‐PI was extremely brittle and therefore its mechanical properties had not been investigated. As shown in **Figure** **2**a, the calculation results showed that the binding energy of H‐bonds formed by different groups varies from 6.5 to 9.0 kJ mol^−1^, and most notably, the binding energy between Cu(I) ions and phenanthroline ligands was as high as 179.6 kJ mol^−1^. As for RFIP‐2, under the action of external mechanical forces, a large number of H‐bonds and Cu(I)‐PDA bonds will break and recombine with the slip of polymer chains, thereby achieving high energy dissipation, which is expected to play an important role in improving the mechanical properties of RFIPs. In other words, the dissociation and association of Cu(I)‐PDA bonds enables the material to dissipate more energy during stretching, which is a good demonstration that RFIP‐2 exhibits the best mechanical properties.

All sample chemical structures were characterized and confirmed by ^1^H NMR and attenuated total reflection‐Fourier transform infrared (ATR‐FTIR) spectroscopy (Figure 2b; Figures S3–S6, Supporting Information). A peak corresponding to the B‐F stretching vibration appeared at 1060 cm^−1^, suggesting the introduction of Cu(I) ions.^[^ ^15^ ^]^ There are no characteristic peaks of the ‐NCO group near 2260–2270 cm^−1^, proving that flexible PU chains were successfully synthesized.^[^ ^16^ ^]^ Significantly, a characteristic peak belonging to the C═O group in the imide group was detected near 1780 cm^−1^ in the RP‐PI spectrum, strongly affirming the successful imidization of rigid PI chains.^[^ ^17^, ^18^ ^]^ Characterization by gel permeation chromatography (GPC) yielded that the number average molecular weight (*M*_n_) of five polymers ranged from 44 to 112 kDa and the polydispersity index (*M*_w_/Mn) was ≈1.4 to 1.6 (Figure S7 and Table S2, Supporting Information). X‐ray diffraction (XRD) curves showed a broad hump with no obvious diffraction peak, indicating their amorphous nature (Figure 2c). Their thermal properties were evaluated by differential scanning calorimetry (DSC) and thermogravimetric analysis (TGA). DSC results (Figure 2d) showed that the glass transition temperature (*T*_g_) of the soft segments in FP‐PU and FIP‐PU were determined to be −37 °C, highlighting the excellent flexibility of the molecular chains at ambient temperature; as the incorporation amount of PI chains increased, a new step appeared near 15 °C (corresponding to the RP‐PI in Figure S8, Supporting Information), which was attributed to the greater intermolecular interaction force generated by woven cross‐linking. Additionally, TGA results (Figure 2e_1_ and e_2_) revealed that all polymers demonstrate high stability with an initial decomposition temperature of ≈280 °C, which can meet diverse practical application needs.

### 2.2 Mechanical Properties

**Figure 3:**
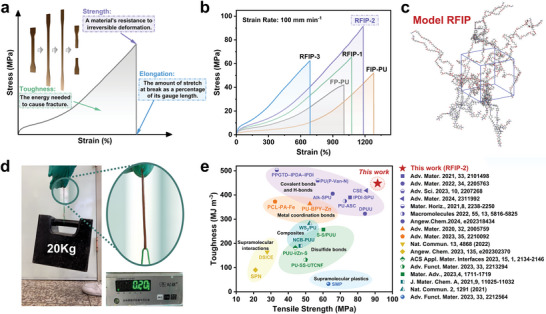
Mechanical properties of RFIPs. a) Definitions of strength, toughness, and elongation. b) Typical stress–strain curves of all polymers. c) Snapshots showing the MD simulations of the optimized Model RFIP. d) Photographs showing a rectangular RFIP‐2 specimen (weight: 0.20 g) lifting a weight of 20.0 kg. e) Ashby plots of toughness versus tensile strength for some recently reported high‐performance, self‐healable poly(urethane‐urea), PU, or supramolecular elastomers.

In uniaxial tensile testing, as depicted in **Figure** **3**a, strength indicates the resistance of a material to irreversible deformation, toughness represents the energy needed to cause the material to fracture, and elongation denotes the amount of stretch at the break as a percentage of its gauge length; in short, the mechanical properties of a material are assessed by a combination of these factors. Figure 3b shows the typical engineering stress–strain curves and **Table** **1** summarizes the mechanical properties of all polymers. From the tensile stress–strain curves, the FP‐PU shows a tensile strength (i.e., ultimate engineering stress) of 42.0 ± 2.4 MPa, an elongation of 1005 ± 71.4%, and a toughness of 178.4 ± 26.3 MJ m^−3^; in stark contrast, the FIP‐PU has higher strength (52.0 ± 0.8 MPa), elongation (1283 ± 33.7%), and toughness (201.3 ± 10.4 MJ m^−3^). Subsequently, the rigid PI and Cu(I) ions were integrated into the PU network, with flexible PU and rigid PI chains being woven together, yielding RFIP‐2 featuring an impressive tensile strength of 91.4 ± 3.3 MPa (twice that of FP‐PU), Young's modulus of 23.1 ± 0.5 MPa, an elongation at break of 1185 ± 3.5%, and toughness as high as 448.0 ± 14.2 MJ m^−3^. However, an excess of PI chains (i.e, RFIP‐3) would lead to an increase in modulus to 44.9 ± 2.0 MPa, accompanied by a significant reduction in elongation to 688 ± 10.5%, and a decrease in toughness to 209.4 ± 9.2 MJ m^−3^. Based on this, the reasonable weaving among PI, PU chains, and Cu(I) ions within molecular networks can enable “rigid and flexible” networks in the true sense, achieving the optimal balance between mechanical strengthening and toughening. In addition to the binding energies of various H‐bonds and metal coordination bonds as evidence, the results of MD simulations also confirmed this view, because the Model RFIP had a larger cohesive energy of 768.65 kJ mol^−1^, while the Model FP‐PU was only 616.56 kJ mol^−1^ (Figure 3c, detailed calculations are provided in the Supporting Information). Moreover, the remarkable mechanical robustness of RFIP‐2 was further affirmed using a rectangular film (≈0.20 g) to lift a weight of 20.0 kg (Figure 3d; Movie S1, Supporting Information). Figure 3e summarized a plot of tensile strength versus toughness of various PU, PUU, or composite systems for comparison, and it is obvious that RFIP‐2 is the first Cu(I)‐based molecularly interwoven polymer involving macromolecular chain weaving (PU and PI chains) reported so far, showing great advantages in mechanical strength and toughness.^[^ ^19^, ^20^, ^21^, ^22^, ^23^, ^24^, ^25^, ^26^, ^27^, ^28^, ^29^, ^30^, ^31^, ^32^, ^33^, ^34^, ^35^ ^]^

**Table 1:** Summary of the mechanical properties of the different polymers, measured by the tensile tests at the strain rate of 100 mm min^−1^.

Sample	Ultimate engineering stress [MPa]	Elongation at Break [%]	Toughness [MJ m^−3^]	Young's modulus [MPa]
FP‐PU	42.0 ± 2.4	1005 ± 71.4	178.4 ± 26.3	19.9 ± 0.3
FIP‐PU	52.0 ± 0.8	1283 ± 33.7	201.3 ± 10.4	16.6 ± 0.8
RFIP‐1	65.6 ± 4.3	1077 ± 20.3	290.1 ± 21.5	22.5 ± 0.5
RFIP‐2	91.4 ± 3.3	1185 ± 3.9	448.0 ± 14.2	23.1 ± 0.5
RFIP‐3	62.3 ± 1.6	688 ± 10.5	209.4 ± 9.2	44.9 ± 2.0

### 2.3 Cyclic Stretch

**Figure 4:**
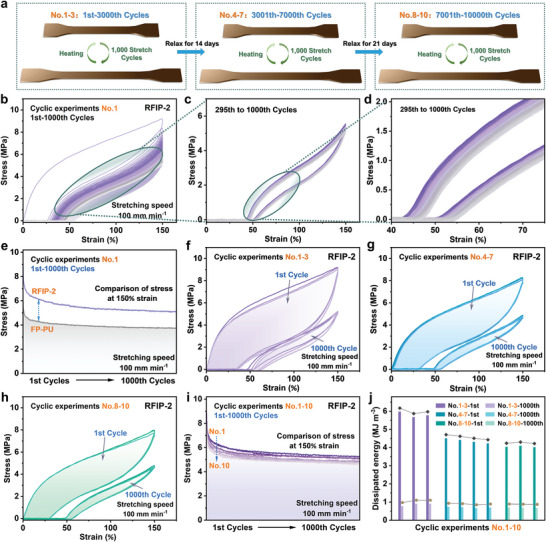
Cyclic stretches of RFIP‐2 and FP‐PU. a) Schematic diagram of all cyclic experiments, divided into three stages from Stage I to Stage III. b) Load‐unload cyclic curves of RFIP‐2 in the cyclic experiment No.1. c,d) Load‐unload cyclic curves for 295th‐1000th cycles of RFIP‐2 in the cyclic experiment No.1. e) Stress comparison of RFIP‐2 and FP‐PU at a fixed strain of 150% from 1st to 1000th cycles. f–h) Comparison of cyclic curves for the 1st cycle and 1000th cycle of cyclic experiments No.1 to No.10. i) Stress comparison of RFIP‐2 at a fixed strain of 150% from 1st to 1000th cycles in cyclic experiments No. 1 to No. 10. j) Comparison of the energy dissipation values of experiments No. 1 to No. 10 at the 1st and 1000th cycles.

To evaluate whether RFIP‐2 could meet the application requirements in engineering environments and highlight its fatigue resistance, we conducted an innovative test with a total of 10 000 tensile cycles at a tensile speed of 100 mm min^−1^ and a fixed strain of 150%. As demonstrated in **Figure** **4**a, it was mainly divided into three stages from Stage I to Stage III. Stage I consisted of three cyclic experiments, numbered cyclic experiments No. 1, No. 2, and No. 3, respectively. Particularly, every 1000 consecutive cycles were recorded as one cyclic experiment, and the tested sample needed to be heated back to its original length before proceeding to the next cyclic experiment. This sample was then placed under ambient conditions for 14 days to undergo Stage II, which consisted of four cyclic experiments for a total of 4000 cycles of tensile testing, denoted as cyclic experiments No. 4 to No. 7. Next, this sample was again placed under ambient conditions for 28 days before proceeding to Stage III, which also consisted of three cyclic experiments, identical to the testing process of Stage I, and was designated cyclic experiments No. 8‐No. 10. As shown in Figure 4b and Movie S2 (Supporting Information), the 1st cycle of RFIP‐2 in No. 1 showed a pronounced hysteresis loop (dissipated energy: 5.97 MJ m^−3^) and residual strain (≈31%), and the resulting large hysteresis proved the occurrence of energy dissipation during stretching. Immediately following, the next cycle without rest had a significantly narrower hysteresis loop that tapered off as the cycle increased, resulting from the untimely reorganization of partially dissociated metal‐ligand bonds (Cu(I)‐PDA) and H‐bonds. After about 295 cycles, the loop ring stabilized without significant shrinkage, implying Cu(I)‐PDA and H‐bonds reached a dynamic equilibrium between breaking and associating (Figure 4c,d). To confirm this, we compared the differences in hysteresis rate and stress between FP‐PU and RFIP‐2 under the same test conditions. The 1^st^ cycle of FP‐PU also exhibited a large hysteresis loop (dissipation energy: 3.68 MJ m^−3^) and residual strain (≈40%), which originates from the large energy dissipation from force‐induced H‐bond breakage (Figure 4e; Figure S9, Supporting Information). In comparison, RFIP‐2 exhibited better tensile strength and energy dissipation capabilities since only H‐bonds and not Cu(I) ions were involved in FP‐PU.

**Figure 5:**
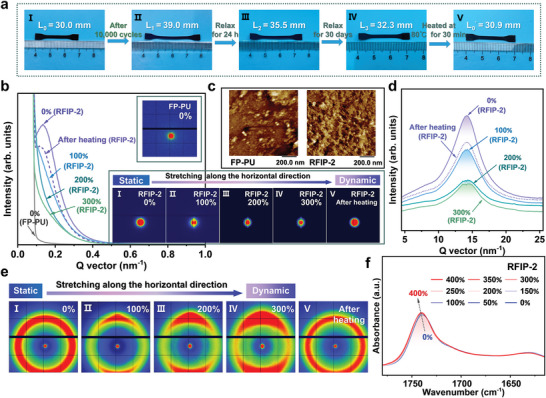
Microphase‐separated structures. a) Photographs of the length of RFIP‐2, after 10000 cycles, relaxing for 24 h, relaxing for 30 days, and heating at 80 °C for 30 min. b) 2D SAXS patterns and 1D profiles of FP‐PU at strain of 0% and RFIP‐2 at strains of 0% (I), 100% (II), 200% (III), 300% (IV), and after heating at 80 °C (V). c) AFM phase images of RFIP‐2 and FP‐PU. d,e) 1D WAXS profiles (d) and 2D WAXS patterns (e) of RFIP‐2 at strains of 0% (I), 100% (II), 200% (III), 300% (IV), and after heating at 80 °C (V). f) Stretch‐related 2D FTIR spectra of RFIP‐2 ranging from 0% to 400%.

After heating at 80 °C for 30 min until RFIP‐2 returned to its original length, cyclic experiments No. 2–10 were carried out in sequence as required (Figure 4f–h; Figures S10–S12, Supporting Information). Figure 4i summarizes the change in stress values at the maximum strain in 10‐cycle experiments, confirming the high elasticity and stress tolerance of RFIP‐2. Figure 4j compared the energy dissipation values of experiments No. 1 to No. 10 at the 1st and 1000th cycles, indicating that RFIP‐2 performed excellent in terms of fatigue resistance and reliability. Intriguingly, after 10 000 cycles, the elongated RFIP‐2 was exposed to ambient conditions and we observed its self‐recovery, with the elongation recovery rate of RFIP‐2 being 38.9% after 24 h of relaxation, 74.4% after 30 days, and reached 90% after heating at 80 °C for 30 min (**Figure** **5**a). In other words, the polymeric material we prepared not only exhibits excellent fatigue resistance and ultra‐high elastic recovery ability under high cyclic loads but also maintains good stability under environmental conditions, with no obvious changes even if left for more than 100 days.

### 2.4 Insights into Strengthening Toughening Mechanisms

#### 2.4.1 Microphase‐Separated Structures

To gain deeper knowledge about the internal structure of woven networks, characterizations were performed at the microscopic level. Small‐angle and wide‐angle X‐ray scattering (SAXS and WAXS) techniques were used to track the structural changes of woven networks from static to dynamic modes during stretching. According to the 2D SAXS patterns and 1D curves (inset in Figure 5b), we observed a scattering peak of RFIP‐2 at q ≈ 0.13 nm^−1^ in static mode, confirming the existence of well‐defined phase‐separated structures with a long period (representing the average distance between hard domains, calculated by Equation 1) of ≈48.3 nm; in contrast, however, FP‐PU did not show clear phase‐separated structures. Atomic force microscope (AFM) phase images also confirmed that RFIP‐2 showed sharper phase‐separated structures, consisting of soft (dark areas) and hard domains (bright areas), but those of FP‐PU were not obvious (Figure 5c; Figure S13, Supporting Information). Predictably, the incorporation of Cu(I) ions in RFIP‐2 transformed the loosely packed H‐bonded cross‐links into tightly packed metal‐ligand and H‐bonded cross‐links and the rigid PI chains brought many rigid structures, thus forming distinguishable microphase‐separated structures. Based on the huge difference in mechanical properties between RFIP‐2 and FP‐PU, it can be surmised that the presence of phase‐separated domains may contribute to the strengthening and toughening of polymers. Accordingly, RFIP‐2 was further examined by SAXS under different tensile strains to reveal the stretch‐induced microstructural evolution (Figure 5b). As the strain increased from 0% to 300%, the initial microphase‐separated structures of RFIP‐2 were disrupted owing to the dissociation and reconstruction of H‐bonds and Cu(I)‐PDA bonds, as evidenced by the disappearance of the diffraction peak, the decrease in the diffraction intensity, and the gradual change of the isotropic scattering ring from circular to rhombic. After the external force was removed, RFIP‐2 was heated back to its original length, accompanied by the re‐formation of the microphase‐separated structures, the reappearance of isotropic scattering rings, and the occurrence of the scattering peak at q ≈ 0.12 nm^−1^ with a long period of 52.4 nm. The above further confirms that the hard domains in the networks change during stretching to allow rapid dissociation and recombination of dynamic bonds, thereby promoting the regeneration of microphase‐separated structures.

Likewise, 2D WAXS results indicated that RFIP‐2 featured an amorphous structure, consistent with previous XRD results; in addition, when the strain increased from 0% to 300%, the isotropic signal ring gradually weakened in the stretching direction and strengthened in the vertical direction, and then heated, the ring returned to isotropy (Figure 5d,e). That is, the hard domains were oriented along the tensile axis with a small amount of crystallite growth that recovered after unloading, suggesting that RFIP‐2 behaved similarly to a classical elastomer. Changes in H‐bonds in the hard domains induced by dynamic uniaxial stretching were well confirmed by stretch‐related 2D FTIR (Figure 5f; Figures S14 and S15, Supporting Information). Combined with Nado's law,^[^ ^36^, ^37^, ^38^ ^]^ the absorption peak at 1737 cm^−1^ (corresponding to the H‐bonded C═O stretching vibration of the ester carbonyl group) shifted to 1755 cm^−1^ (corresponding to the free C═O stretching vibration of the ester carbonyl group) with increasing strains, signifying that the H‐bonded C═O groups were continuously broken and free H‐bonds were gradually formed.

#### 2.4.2 Energy Dissipation

**Figure 6:**
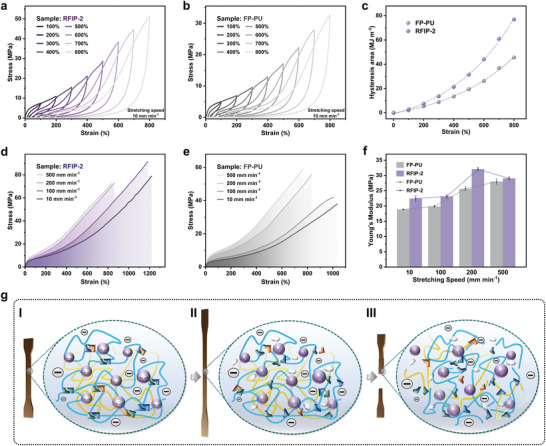
Energy dissipation. a) Load‐unload cycle curves of RFIP‐2 with the strain increasing from 100% to 800% without delay time. b) Load‐unload cycle curves of FP‐PU with the strain increasing from 100% to 800% without delay time. c) Strain‐dependent functions correspond to the hysteresis area of RFIP‐2 and FP‐PU at different strains. d) Tensile stress–strain curves of RFIP‐2 stretched at different speeds. e) Tensile stress–strain curves of FP‐PU stretched at different speeds. f) Young's modulus of RFIP‐2 and FP‐PU at different tensile speeds. g) Schematic diagram of the changes in the RFIP networks during tensile deformation until fracture.

To illustrate the dissipative capabilities of Cu(I)‐PDA bonds, H‐bonds, and woven networks, cyclic stretching of RFIP‐2 and FP‐PU under increasing strain were compared (**Figure** **6**a,b), and the hysteresis area for each loading‐unloading cycle was plotted as a function of strain (Figure 6c). When the load strains of RFIP‐2 were 100%, 400%, 600%, and 800%, the difference in hysteresis energy was noticeable, and the dissipated energies were 2.8, 21.3, 44.1, and 76.9 MJ m^−3^, respectively. Differently, the hysteresis energy variation of FP‐PU was smaller and its dissipated energy was only 45.5 MJ m^−3^ when the load strain reached 800%. The gap in the dissipated energy proves that FP‐PU dissipates load energy primarily through the breaking of urethane and urea H‐bonds, while RFIP‐2 dissipates load energy through more energy‐dissipating groups, including woven networks, H‐bonds, and Cu(I)‐PDA bonds; in other words, RFIP‐2 offers higher load‐bearing capacity and greater resistance to fracture. Upgrading from FP‐PU to RFIP‐2, the mechanical properties of the material are simultaneously strengthened and toughened. Further, the proposed comprehensive energy dissipation mechanism of Cu(I)‐PDA bonds and H‐bonds was also experimentally verified, and tensile tests were carried out at different strain rates from 10 to 500 mm min^−1^, using FP‐PU as a control. The fracture stress, Young's modulus, and toughness of RFIP‐2 and FP‐PU increased but the elongation decreased with improving tensile speed (Figure 6d–f), which strongly confirmed that dynamic non‐covalent bonds (i.e., Cu(I)‐PDA bonds or H‐bonds) exerted an essential role. The increase in strain rate resulted in a shorter time for the broken Cu(I)‐PDA and H‐bonds to re‐form, thereby reducing the fracture tolerance (i.e., the elongation decreases).

Combining the results of MD simulations and experimental analysis, the strengthening toughening mechanisms of the material during the stretching process were proposed (Figures S16 and S17, Supporting Information). First, benefiting from the joint effects of rigid PI and flexible PU chains, Cu(I)‐PDA bonds, and H‐bonds, the RFIP initially displays distinct microphase‐separated structures (Figure 6g‐I). Upon being subjected to an external force, woven networks are first stretched, and then the weaker H‐bonds dissociate first as the RFIP slowly deforms; subsequently, as the external force increases, the stronger H‐bonds and Cu(I)‐PDA bonds also dissociate sequentially, causing the Cu(I) ions on the force‐bearing chains to detach from the coordination binding sites (Figure 6g‐II). Eventually, woven networks become loosened and a large number of free H‐bonds, Cu(I) ions, and PDA ligands are regenerated within the polymer matrix, which effectively suppresses localized stress over‐concentration and improves the ability of load absorption and dissipation (Figure 6g‐III). After undergoing the evolution of the microstructures described above, RFIP shows higher strength, toughness, and stretchability.

### 2.5 Shape Memory

**Figure 7:**
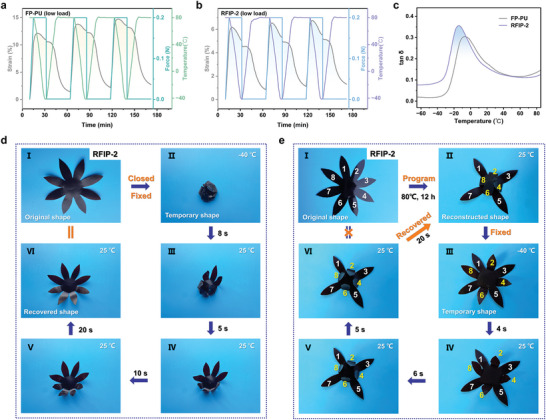
a) Shape memory cycle DMA curves of FP‐PU under a load of 0.2N. b) Shape memory cycle DMA curves of RFIP‐2 under a low load of 0.2N. c) The tan δ of FP‐PU and RFIP‐2. d) Photographs to demonstrate the shape fixing and recovery process of RFUP‐2. e) Photographs to illustrate the shape‐reconstruction and shape‐memory process of RFUP‐2.

Dynamic mechanical analysis (DMA) quantitatively investigated the shape memory properties of RFIP‐2 and FP‐PU under different loads (1.0 N and 0.2 N) and recorded their storage modulus (Eʹ) and loss factor (tan δ) (**Figure** **7**a–c; Figures S18–S20, Supporting Information). All results show larger unrecovered strains in the first cycle, probably due to the plastic deformation of the molecular chains after being heated above *T*_g._ However, as the number of cycles increases (until the 3rd cycle), under a load of 1.0 N, the shape recovery rates (*R*_r_) of FP‐PU and RFIP‐2 increased from 86.7% to 97.5% and 87.6% to 96.6%, respectively; similarly, under a load of 0.2N, the *R*_r_ of FP‐PU and RFIP‐2 increased from 87.0% to 95.1% and 83.3% to 95.5%, respectively. These might be attributed to the increased compliance of the molecular chains and the reduced entanglement of the chains in the other direction as the number of cycles increases. On average, both FP‐PU and RFIP‐2 exhibited high shape fixation rates (*R*_f_) at both high and low loads (Table S3, Supporting Information). Hence, shape memory behaviors under different loads showed stable shape fixation and recovery, making the elucidation of the shape memory effect more illustrative. Here, our study focuses specifically on the one‐way shape memory effect of the material, which is the ability to recover from a temporary shape to its original shape only under specific conditions after undergoing deformation. The favorable one‐way shape memory effect of the material was demonstrated by a heating‐cooling process, in detail, by bending or closing RFIP‐2 and FP‐PU with various shapes to form temporary shapes at low temperatures, and then the temporary shapes could be restored to shapes that were virtually indistinguishable from the original shapes at room temperature in a short time (Figure 7d; Figures S21 and S22, Movies S3 and S4, Supporting Information). Intriguingly, since shape memory polymers can directly output displacements and forces when phase transitions occur, both RFIP‐2 and FP‐PU can achieve shape memory‐driven advancement or movement (Movie S5, Supporting Information); meanwhile, RFIP‐2 displays faster shape response than FP‐PU due to the uniform distribution of rigid PI, flexible PU chains, and Cu(I) ions, which is promising for applications in the field of intelligent robotics and temperature signal transmission in extremely cold environments. That is, at extremely low temperatures, they can also play the role of temporary shapes to perform some tasks or functions.

Also, originating from the synergistic action of dynamic Cu(I)‐PDA bonds and H‐bonds, the initial shape of RFIP‐2 can be reshaped when the molecular chains unwind and exchange. Initially, each petal of the “sunflower”‐shaped RFIP‐2 was numbered in sequence, the #2, #4, #6, and #8 petals were folded inward and shaped, and then placed in an 80 °C oven for 12 h to program a new permanent shape (Figures 7e‐I,II). Subsequently, take out the programmed RFIP‐2, unfold all the petals, and place them in liquid nitrogen at −40 °C to fix the temporary shape (Figure 7e‐III). Ultimately, the programmed petals #2, #4, #6, and #8 gradually closed and recovered to their programmed shape at 25 °C, as shown in (Figure 7e‐IV–VI). Accordingly, the generated rigid‐flexible polymers have exceptional shape memory and reconfiguration capabilities and can achieve precisely controlled and repeatable shape changes, showing great application potential in high‐end applications such as intelligent robotics.

## 3 Conclusion

To cope with the inherent contradiction between strength and toughness in pursuit of superior high‐performance polymeric materials, we started by exploring the evolution of polymer chains at the microscale to realize macro‐control of the material's mechanical properties. Inspired by “molecular weaving”, we report for the first time a new RFIP with remarkable mechanical properties (strength: 91.4 ± 3.3 MPa; toughness: 448.0 ± 14.2 MJ m^−3^), fatigue resistance (recoverable after 10 000 cyclic stretches), and shape memory properties, consisting of flexible PU and rigid PI chains cleverly woven together around the Cu(I) ions center. The fatigue‐resistant, ultra‐strong shape memory polymeric materials prepared in this work can be employed in high‐end equipment (such as TBMs), signal transmission, and flexible electronic products in the future. In brief, we have fused the essence of weaving on the atomic and molecular levels to obtain brilliant and valuable mechanical properties, offering new perspectives in designing robust and stable polymers.

## Conflict of Interest

The authors declare no conflict of interest.

## Supporting information

Supporting Information

Supplemental Movie 1

Supplemental Movie 2

Supplemental Movie 3

Supplemental Movie 4

Supplemental Movie 5

## Data Availability

The data that support the findings of this study are available from the corresponding author upon reasonable request.
