# Supplementary material for: Super‐Durable, Tough Shape‐Memory Polymeric Materials Woven from Interlocking Rigid‐Flexible Chains
Source: Adv Sci (Weinh). 2024 Aug 5;11(38):2406193. doi: 10.1002/advs.202406193 (PMC11481217; doi:10.1002/advs.202406193)
Supplement: Supplementary file 1 — Supporting Information [file ADVS-11-2406193-s002.pdf]

ADVANCED SCIENCE Open Access

Supporting Information

for *Adv. Sci.*, DOI 10.1002/advs.202406193

Super-Durable, Tough Shape-Memory Polymeric Materials Woven from Interlocking Rigid-Flexible Chains

Jing Xu, Mingchao Shao, Tianze Chen, Song Li, Yaoming Zhang, Zenghui Yang, Nan Zhang, Xinrui Zhang, Qihua Wang* and Tingmei Wang**

Supporting Information

Super-durable, Tough Shape-memory Polymeric Materials Woven from Interlocking Rigid-flexible Chains

Jing Xu[1,2,3], Mingchao Shao[1,3], Tianze Chen[1,2,3], Song Li[1,3], Yaoming Zhang[1,3], Zenghui Yang[1,3], Nan Zhang[1,3], Xinrui Zhang[1,3,*], Qihua Wang[1,2,3,*], Tingmei Wang[1,2,3,*]

[1] *State Key Laboratory of Solid Lubrication, Lanzhou Institute of Chemical Physics, Chinese Academy of Sciences, Lanzhou 730000, China.*

[2] *Center of Materials Science and Optoelectronics Engineering, University of Chinese Academy of Sciences, Beijing 100049, China.*

[3] *Key Laboratory of Science and Technology on Wear and Protection of Materials, Lanzhou Institute of Chemical Physics, Chinese Academy of Sciences, Lanzhou 730000, China.*

Corresponding authors (*):

Prof. Xinrui Zhang

Lanzhou Institute of Chemical Physics, Chinese Academy of Sciences, P. R. China

Tel: +86-931-4968369

Fax number: +86-931-4968252

Email: xruiz@licp.cas.cn

Prof. Qihua Wang

Lanzhou Institute of Chemical Physics, Chinese Academy of Sciences, P. R. China

E-mail: wangqh@licp.cas.cn

Prof. Tingmei Wang

Lanzhou Institute of Chemical Physics, Chinese Academy of Sciences, P. R. China

E-mail: tmwang@licp.cas.cn

Table of contents

1. **Materials**
2. **Preparation of RFIPs, FP-PU, FIP-PU, and RP-PI.**

    2.1 Synthesis of flexible PU chains

    2.2 Synthesis of rigid PI chains

    2.3 Synthesis of RFIPs

3. **Experimental Section**

    3.1 General characterizations

    3.2 Wide-angle and small-angle X-ray scattering (WAXS and SAXS) measurements

    3.3 Tensile tests

    3.4 Cyclic tensile tests

    3.5 Shape memory tests

4. **Molecular Dynamics Simulations**

    4.1 Simulations of the structures of Model FP-PU and Model RFIP

    4.2 Calculation of the cohesive energy

    4.3 Simulation of tensile properties

5. **Supporting Figures and Tables**

    Figure S1. Synthetic routes of flexible PU chains.

    Figure S2. Synthetic routes of rigid PI chains.

    Figure S3. ^{1}H NMR spectra of FP-PU recorded in $CDCl_3$.

    Figure S4. 1H NMR spectra of FIP-PU recorded in CDCl3.

    Figure S5. ^{1}H NMR spectra of RFIP-2 recorded in $CDCl_3$.

    Figure S6. ATR-FTIR spectra of RP-PI.

    Figure S7. GPC traces were recorded for FP-PU, FIP-PU, and RFIPs by using DMF as the elution solvent.

    Figure S8. DSC curves of RP-PI.

    Figure S9. Load-unload cyclic curves of FP-PU in the cyclic experiment **No.1**.

    Figure S10. Load-unload cyclic curves of RFIP-2 in the cyclic experiment **No.1-No.3**.

Figure S11. Load-unload cyclic curves of RFIP-2 in the cyclic experiment **No.4-No.7**.

Figure S12. Load-unload cyclic curves of RFIP-2 in the cyclic experiment **No.8-No.10**.

Figure S13. AFM phase images of FIP-PU, RFIP-1, and RFIP-3.

Figure S14. Stretch-related ATR-FTIR spectra of RFIP-2 in the 3600~900 cm^{-1} band.

Figure S15. The synchronous and asynchronous 2D-COS spectra for the 1800~1500 cm-1 band of RFIP-2. Red and blue represent positive and negative, respectively.

Figure S16. Snapshots showing the MD simulations of Model FP-PU at strains of 0%, 100%, 400%, and 800%.

Figure S17. Snapshots showing the MD simulations of Model RFIP at strains of 0%, 100%, 400%, and 800%.

Figure S18. The Storage Modulus (E′) of RFIP-2 and FP-PU.

Figure S19. Shape memory cycle DMA curves of RFIP-2 under a high load of 1.0N.

Figure S20. Shape memory cycle DMA curves of FP-PU under a high load of 1.0N.

Figure S21. Photographs showing the process of shape fixation and recovery of RFIP-2 and FP-PU.

Figure S22. Photographs showing the process of shape fixation and recovery of the "sunflower" shaped FP-PU.

Table S1a. Detailed synthesis parameters for all polymers.

Table S1b. Detailed synthesis parameters for all polymers.

Table S2. Physical properties parameters of all polymers.

Table S3. Summary of shape fixation rates (R_f) and shape recovery rates (R_r) for FP-PU and RFIP-2 under different loads.

6. Supporting Movies

Movie S1. A rectangular RFIP-2 film can easily lift a weight of 20.0 kg

Movie S2. Cyclic tensile testing of RFIP-2 from 1st to 300th cycle.

Movie S3. Shape memory-driven forward or motion process of RFIP-2 and FP-PU.

Movie S4. Shape memory process of the rectangular RFIP-2 and FP-PU at 25 °C.

Movie S5. Shape memory process of the flower-shaped RFIP-2 and FP-PU at 25 °C.

7. Supporting References

1. Materials

Isophorone diisocyanate (IPDI, 99%), Cu(I)tetra(acetonitrile)tetrafluoroborate ($Cu(MeCN)_4BF_4$), dibutyltin dilaurate (DBTDL, 98%), N-methyl pyrrolidone (NMP), N,N-dimethylacetamide (DMAc), 4,4'-diaminodicyclohexyl methane (DDCM), and 4,4'-oxydiphthalic anhydride (ODA) were sourced from Energy Chemical Co., Ltd. Polycarbonate diol (PCDL, $M_n = 2000\ g\ mol^{-1}$) was purchased from Jining Liduo Chemical Co., Ltd. And 4,4'-(1,10-phenanthroline-2,9-diyl)dianiline (PDA) was purchased from Shanghai Tensus Biotech Co., Ltd. All other chemicals and solvents were analytical reagents and used without further purification.

2. Preparation of RFIPs, FP-PU, FIP-PU, and RP-PI.

2.1 Synthesis of flexible PU chains

In advance, PCDL-2000 was placed in a three-necked flask and stirred for 2 hours at 120°C under a nitrogen atmosphere to remove residual water. In the first step, when the mixture was cooled to 80°C, excess IPDI, a small amount of DBTDL catalyst, and organic solvent DMAc were added into the reaction flask, stirred for 3 hours, and then a PU prepolymer was generated. In the second step, DDCM was subjected to a chain extension reaction with the PU prepolymer, and the reaction was carried out under a nitrogen atmosphere for about 1 hour. In the third step, add another amine chain extender, PDA, and continue heating and stirring for about 1 hour to further generate the required flexible PU chains.

FP-PU: Poured the synthesized flexible PU chains into a Teflon mold and vacuum-dried at 80 °C to obtain a dry film, named FP-PU. The amounts of each monomer of FP-PU are shown in **Tables S1a and 1b**.

FIP-PU: $Cu(MeCN)_4BF_4$ was added to the synthesized flexible PU chains and stirred for about 3 hours under a nitrogen atmosphere at 50°C to obtain flexible interlocking PU chains. Finally, the solution was poured into a Teflon mold and vacuum-dried at 80 °C to obtain a dry film, named FIP-PU. The amounts of each monomer of FIP-PU are shown in **Tables S1a and 1b**.

2.2 Synthesis of rigid PI chains

Initially, the ODA solution dissolved in NMP solvent was added to a three-neck 250 mL round-bottom flask equipped with a magneton, Dean-Stark trap, water condenser, and nitrogen inlet. In the first step, PDA was slowly added to the ODA solution in batches, and the reaction solution was further stirred with a magnetic stirrer under a nitrogen atmosphere and an ice bath for about 24 hours. In the second step, a certain amount of toluene (1/10 volume of NMP) was added to the reaction device, and then the reaction solution was heated at 230°C for 1 hour using a Dean-Stark apparatus to remove water.

RP-PI: Poured the synthesized rigid PI chains into a Teflon mold and vacuum-dried at 80 °C to obtain a dry polymer, named RP-PI. The amounts of each monomer of RP-PI are shown in **Tables S1a and 1b**.

2.3 Synthesis of RFIPs

Poured the synthesized flexible PU and rigid PI chains into a three-necked flask in sequence, then added $Cu(MeCN)_4BF_4$ and stirred for about 3 hours under a nitrogen atmosphere at 50°C to achieve the weaving of PI and PU chains. Finally, the solution was poured into a Teflon mold and vacuum-dried at 80 °C for 48 hours to obtain a dry film. The added amounts of each monomer for different samples are shown in **Tables S1a and 1b**, and the samples are named RFIP-1, RFIP-2, and RFIP-3, respectively.

3. Experimental Section

3.1 General characterizations

Hydrogen proton nuclear magnetic resonance (^1H NMR) spectra were measured on an Avance NEO 400 M spectrometer (Bruker, Germany). Attenuated total reflection Fourier transform infrared (ATR-FTIR) spectra were collected by a Nicolet Nexus 870 spectrometer (Bruker, Germany) with a scan range from 500 to 4000 cm^{-1} in attenuated total reflection mode. Stretch-related 2D FTIR spectra were recorded on an IRAffinity-1S spectrometer (Shimadzu, Japan), equipped with a diamond ATR. The samples were scanned at different temperatures and different strains over a wavelength range of 600 to 4000 cm^{-1} with a resolution of 2 cm^{-1}. The samples were tensile tested using an Instron 3367 tensile machine (Instron, USA) with a tensile rate of 5 mm min^{-1}. 2D FTIR spectra were tested at different strains: 0%, 50%, 100%, 150%, 200%, 250%, 300%, 350%, and 400%. X-ray diffraction (XRD) tests were conducted on Ultima IV (Rigaku, Japan) using Cu-Kα radiation with $\lambda = 1.5418$ Å, a scanning range of $2\theta = 5°$ to 90°, and a scanning rate of 5° min^{-1}. The microstructure of all polymers was observed by a Dimension Icon atomic force microscope (Bruker, Germany), and the corresponding atomic force microscopy (AFM) results were obtained in tapping mode with a scanning area of 1 μm × 1 μm. Thermo-gravimetric analysis (TGA) was performed on an SDT 650 synchronous thermal analyzer (TA, USA) with a temperature range of 25 °C to 850 °C under nitrogen flow (100 mL min^{-1}) at a ramp rate of 10 °C min^{-1}. Differential scanning calorimetry (DSC) was analyzed by DSC STA449 F3 (NETZSCH, Germany) under constant nitrogen flow at a heating rate of 10 °C min^{-1}. The tested samples (weight: 3-5 mg) were subjected to heating-cooling cycles (heating rate: 10 °C min^{-1}): first heating to 200 °C, then cooling to -120 °C and finally re-heating to 200 °C. Rectangular samples were tested on a Q800 Dynamic Mechanical Analyzer (TA, USA) using dynamic temperature sweep mode in the range -100 to 150 °C with a heating rate of 5 °C min^{-1} and a frequency of 1 Hz. The molecular weight information of all samples was determined using PL GPC50 gel permeation chromatography (Agilent, USA) with DMF as the mobile phase.

3.2 Wide-angle and small-angle X-ray scattering (WAXS and SAXS) measurements

WAXS and SAXS measurements were conducted on a Xeuss 3.0 (Xenocs, France) with a semiconductor detector, running with a phototube power of 30 W and generating Cu-Kα radiation with λ=1.5418 Å. Each WAXS data in the center of the sample was acquired within 600 seconds at a sample-to-detector distance of 85.0 mm, as well as the WAXS patterns were background corrected through the standard procedures. In addition, each SAXS data in the center of the sample was acquired within 600 seconds at a sample-to-detector distance of 1000 mm, and the SAXS patterns were background corrected through the standard procedures.

The periodicity (L) was calculated by the Bragg's law (**Eq1**):

$$L = \frac{2\pi}{q_{max}} \quad (\mathbf{Eq1})$$

where q_{max} corresponds to the peak position of the 1D SAXS curve.

3.3 Tensile tests

The stress-strain curves at different tensile speeds at room temperature were recorded by an electronic universal tensile testing machine (Shimadzu AG-X, Japan). All specimens were cut into dumbbell shapes (size: 30 mm × 2 mm × 0.2~0.5 mm) and each specimen was examined three times to obtain a reliable average.

Toughness (τ) can be defined by integrating the area under the stress-strain curve, calculated by the following formula (**Eq2**):

$$\tau = \int_{\varepsilon=0}^{\varepsilon=\varepsilon_{max}} \sigma \, d\varepsilon \quad (\mathbf{Eq2})$$

where σ is the stress value, ε is the strain value, and ε_{max} is the elongation at break.

3.4 Cyclic tensile tests

For the cyclic tensile test, the whole process was implemented by stretching the specimen from 0% to 150% strain and 1000 consecutive cycles at a constant tensile speed of 100 mm min^{-1}. After the cycled specimen was returned to its original length,

the next 1,000-cycle test was performed and repeated 10 times, for a total of 10,000 cycles. Besides, strain-related cyclic tensile experiments were performed in which the specimens were sequentially stretched from 0% to 800% strain without any rest. For a quantitative evaluation of the energy dissipation, the dissipated energy value was calculated from the area of the hysteresis loop.

3.5 Shape memory tests

The shape memory properties of RFIP-2 and FP-PU were characterized using Q800 DMA (TA, USA). A shape memory cycle process is as follows: Each specimen is first heated to 80°C and held for 10 min to eliminate thermal history. Then the specimen is deformed after a load is applied. After reaching the set load, the load is kept constant, the temperature is cooled to -40°C, and the length of the specimen is denoted as ε_m. Keep it for 5 minutes until the material is completely crystallized, then remove the load, and the length of the specimen is recorded as ε_u. Finally, the temperature is again raised to 80°C, and the length of this specimen is recorded as ε_p.

The experiment allowed the determination of the shape fixation rate (R_f), as well as the shape recovery rate (R_r). The R_f quantifies the fixability of the temporary form and the R_r describes to what extent the permanent shape is recovered. According to reported literature[1], the R_r was calculated from ε_m and the extension at the load-free states the (N-1)th cycle ε_p and the Nth cycle ε_p (**Eq3**), and the R_f was given by the ratio of the strain in the load-free state after the retraction of the tensile stress in the Nth cycle ε_u and the maximum strain ε_m (**Eq4**).

$$R_r(N) = \frac{\varepsilon_m - \varepsilon_p(N)}{\varepsilon_m - \varepsilon_p(N-1)} \quad \textbf{(Eq3)}$$

$$R_f(N) = \frac{\varepsilon_u(N)}{\varepsilon_m} \quad \textbf{(Eq4)}$$

4. Molecular Dynamics Simulations

4.1 Simulations of the structures of Model FP-PU and Model RFIP

Molecular dynamic (MD) simulations were carried out to investigate the number of H-bonds and cohesive energies of different polymers. Two different all-atom models named Model FP-PU and Model RFIP respectively, were considered in the simulations. All MD simulations were carried out by the Forcite module with the COMPASS III force field in Materials Studio (MS) 2020.[2] All-atom MD simulation systems are comprised of five polymer chains. In the Model FP-PU, five flexible PU chains were randomly packed into one cubic simulation box, while the Model RFIP contained five rigid PI chains. After energy minimization and 25-circle annealing, each system was subjected to MD simulation under periodic boundary conditions for 1000 ps in the NPT (P = 1 atm, T = 298 K) ensemble using the Nose thermostat and Berendsen barostat, which was long enough for the system temperature, potential and total energy to become stable. After reaching the equilibrium state, another 400 ps simulation under the NVT ensemble was performed to extract the trajectory and data for calculating the number of H-bonds and total cohesive energy. Dynamic traces for each system are output at 4 ps intervals. Moreover, the number of H-bonds in the two included systems was analyzed by written Perl code. The criteria for H-bonds are that the length is less than 2.5 Å and the angle is greater than 120 degrees.

4.2 Calculation of the cohesive energy

The cohesive energy per chain is defined as the average energy per chain required to separate all polymer chains in the condensed state from each other by an infinite distance. MD simulations were also carried out to investigate the cohesive energies of different polymers. Van der Waals and Coulomb interactions were calculated using the atom-based and Ewald methods, respectively, with a cut-off value of 12.5 Å. Equations of motion were integrated with a time step of 1 fs. The cohesive energy per chain was calculated by the following formula (**Eq5**)[3]:

$$E_{cohesive} = \frac{\sum_{i=1}^{5} E_{chain}^{isolated}(i) - E_{pot}^{5}}{5} \quad (\boldsymbol{Eq5})$$

where $E_{chain}^{isolated}$ is the average potential energy of an isolated polymer chain in a vacuum, and E_{pot}^{5} is the average potential energy of the condensed system consisting of five polymer chains. The potential energies of the isolated polymer chains were calculated by averaging the potential energy of a single chain in 10 frames. For E_{pot}^{5}, the potential energies were calculated by averaging the potential energy of the condensed system over the same number of frames during NVT simulation.

For the Model FP-PU, $E_{cohesive}$= 616.56 kJ/mol.

For the Model RFIP, $E_{cohesive}$= 768.65 kJ/mol.

4.3 Simulation of tensile properties

To understand the strengthening toughening mechanisms more vividly, we constructed amorphous cells of Model FP-PU and Model RFIP amorphous units with 3D periodic boundary conditions and simulated the stretching process through MD simulation. The Model FP-PU and Model RFIP configurations obtained from the above simulation are stretched along the X direction. Initially, the cell was subjected to axial tension from the initial stress to produce different strains. The deformed cell was then equilibrated with 1000 steps under the NPT ensemble to obtain the final stretched structure at various strains. All modeling and simulations were performed using the Forcite module with the COMPASS III force field in MS 2020.

5. Supporting Figures and Tables

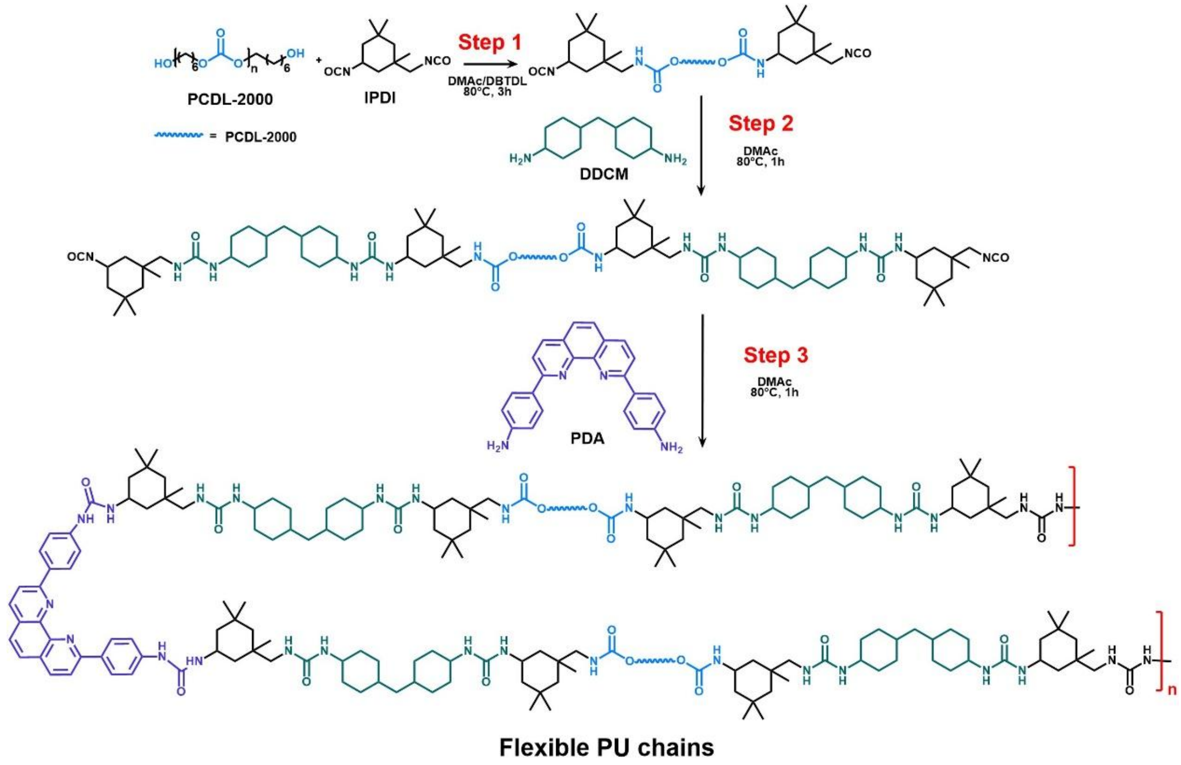

Figure S1. Synthetic routes of flexible PU chains.

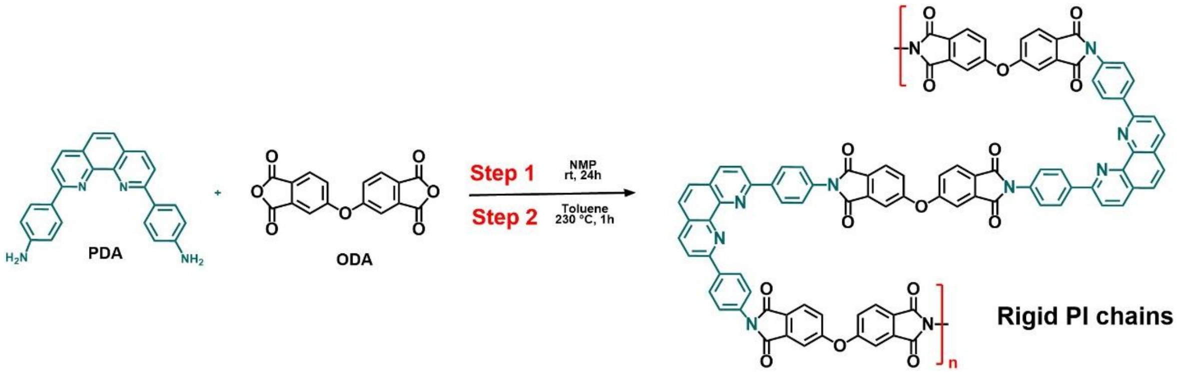

Figure S2. Synthetic routes of rigid PI chains.

Table S1a. Detailed synthesis parameters for all polymers.

Sample	Flexible PU chains				Rigid PI chains		Cu(I) ions
	PCDL (mmol)	IPDI (mmol)	DDCM (mmol)	PDA (mmol)	ODA (mmol)	PDA (mmol)	$Cu(MeCN)_4BF_4$ (mmol)
**FP-PU**	1	2	0.8	0.2	0	0	0
**FIP-PU**	1	2	0.8	0.2	0	0	0.1
**RFIP-1**	1	2	0.8	0.2	0.1	0.1	0.15
**RFIP-2**	1	2	0.8	0.2	0.2	0.2	0.2
**RFIP-3**	1	2	0.8	0.2	0.3	0.3	0.25
**RP-PI**	0	0	0	0	0.1	0.1	0

Table S1b. Detailed synthesis parameters for all polymers.

Sample	Flexible PU chains				Rigid PI chains		Cu(I) ions
	PCDL (mg)	IPDI (mg)	DDCM (mg)	PDA (mg)	ODA (mg)	PDA (mg)	$Cu(MeCN)_4BF_4$ (mg)
**FP-PU**	2000	445	168.3	72.4	0	0	0
**FP-PU**	2000	445	168.3	72.4	0	0	0.1
**RFIP-1**	2000	445	168.3	72.4	31.0	36.2	47.2
**RFIP-2**	2000	445	168.3	72.4	62.0	72.4	62.9
**RFIP-3**	2000	445	168.3	72.4	93.1	108.3	78.6
**RP-PI**	0	0	0	0	31.0	36.2	0

Molecular weight: PCDL: 2000 g/mol; IPDI: 222.32 g/mol; DDCM: 210.36 g/mol; PDA: 362.43 g/mol; ODA: 310.21 g/mol; $Cu(MeCN)_4BF_4$: 314.56 g/mol.

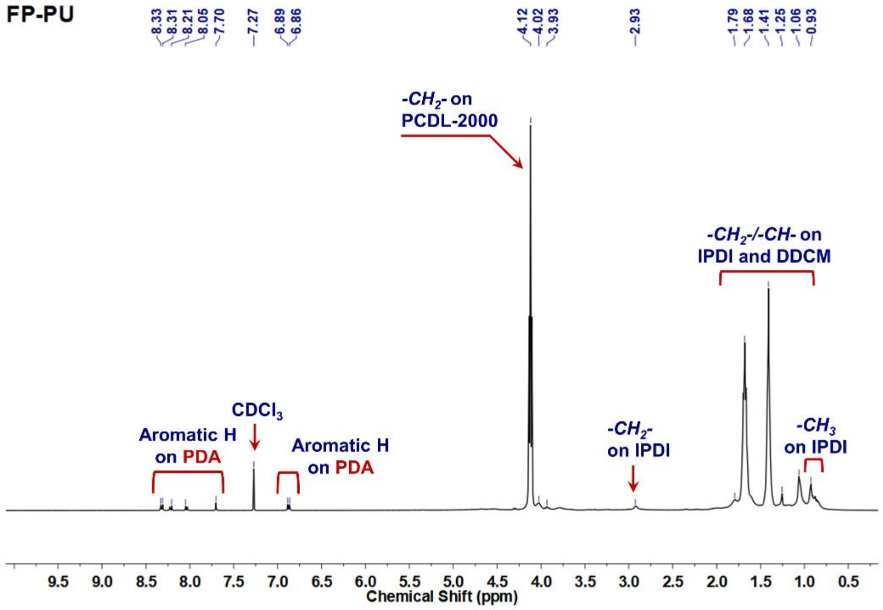

Figure S3. ^{1}H NMR spectra of FP-PU recorded in $CDCl_3$.

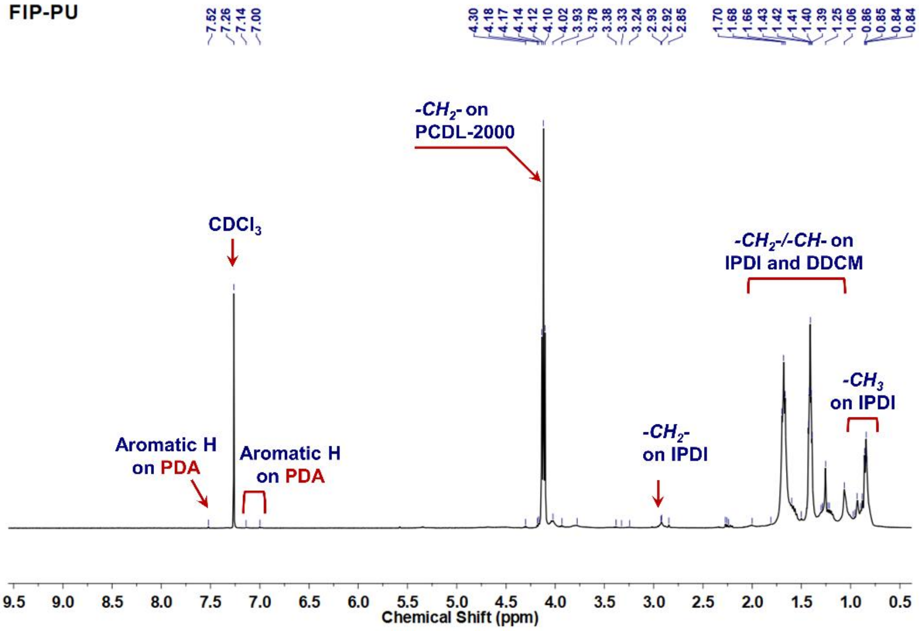

Figure S4. ^{1}H NMR spectra of FIP-PU recorded in $CDCl_3$.

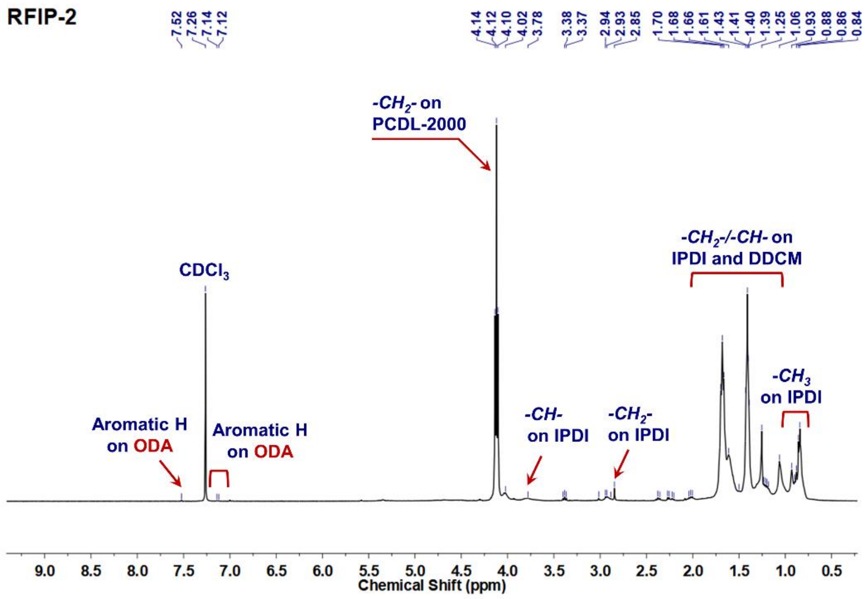

Figure S5. ^{1}H NMR spectra of RFIP-2 recorded in $CDCl_3$.

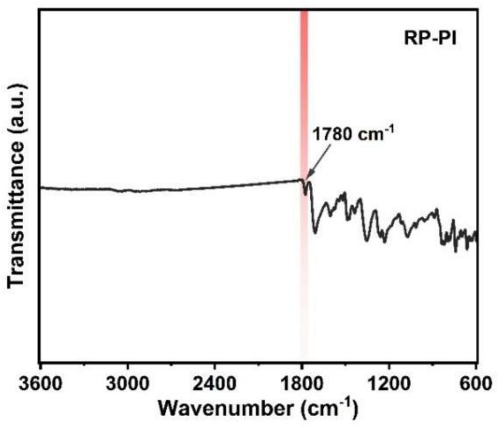

Figure S6. ATR-FTIR spectra of RP-PI.

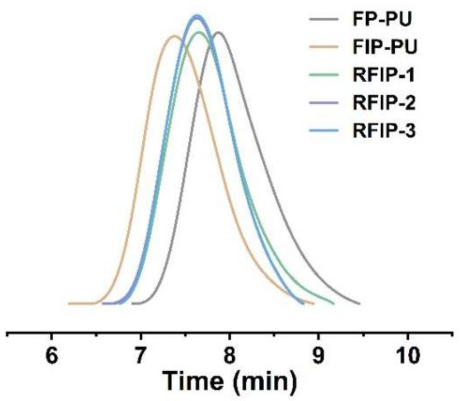

Figure S7. GPC traces were recorded for FP-PU, FIP-PU, and RFIPs by using DMF as the elution solvent.

Table S2. Physical properties parameters of all polymers.

Sample	Mn [a] [Da]	Mw [b] [Da]	PDI [c] [Mw/Mn]
FP-PU	44643	73078	1.64
FIP-PU	112445	177402	1.58
RFIP-1	72395	115384	1.59
RFIP-2	84945	123588	1.45
RFIP-3	84522	121855	1.44

[a] Number-average molecular weight.

[b] Weight-average molecular weight.

[c] Polydispersity index.

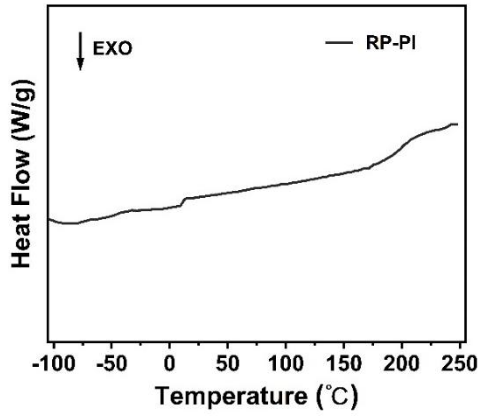

Figure S8. DSC curves of RP-PI.

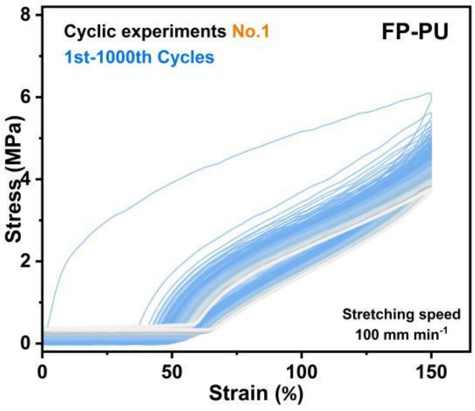

Figure S9. Load-unload cyclic curves of FP-PU in the cyclic experiment No.1.

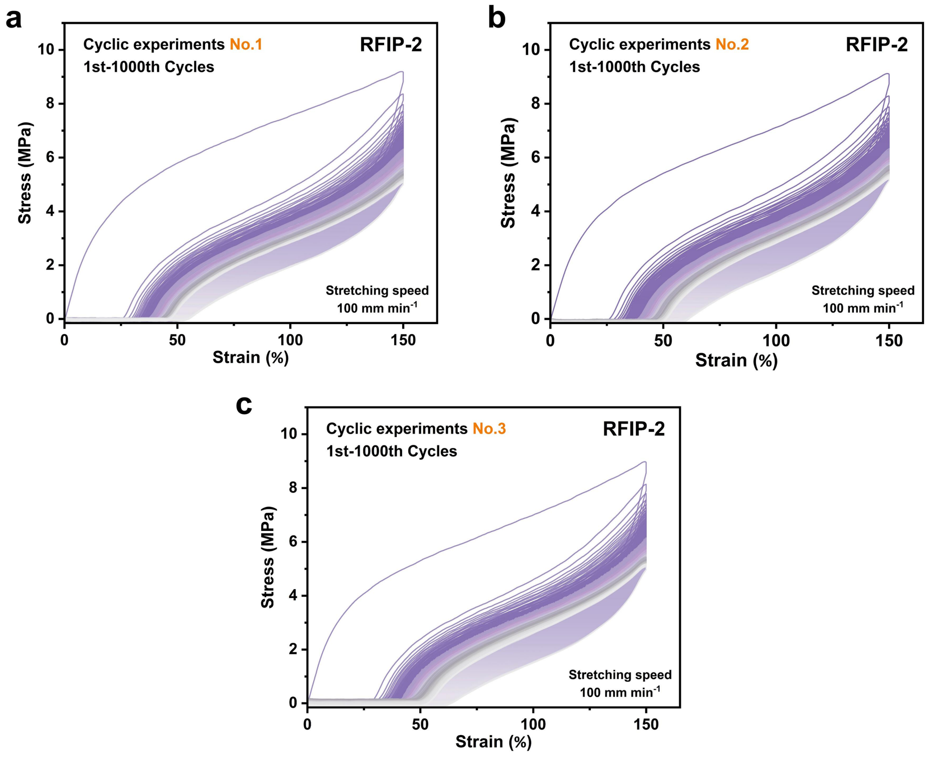

Figure S10. Load-unload cyclic curves of RFIP-2 in the cyclic experiment No.1-No.3.

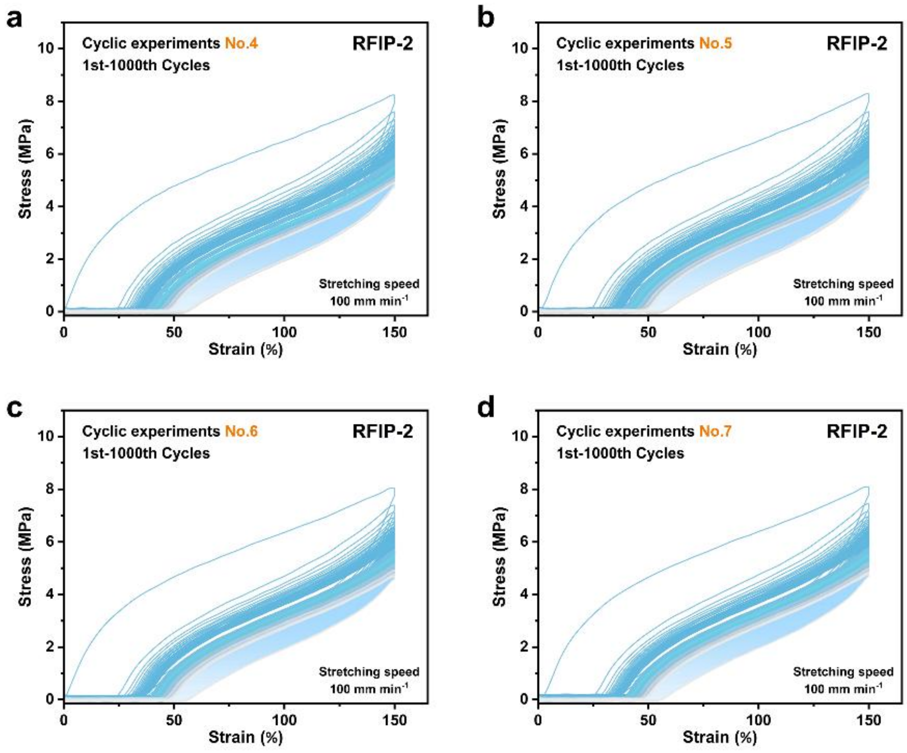

Figure S11. Load-unload cyclic curves of RFIP-2 in the cyclic experiment No.4-No.7.

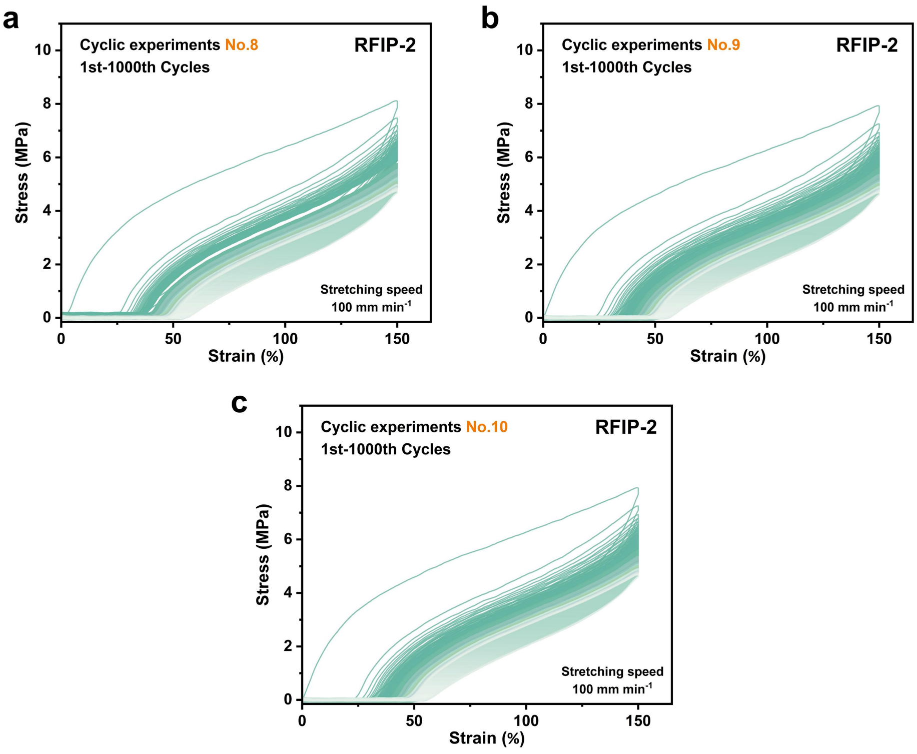

Figure S12. Load-unload cyclic curves of RFIP-2 in the cyclic experiment No.8-No.10.

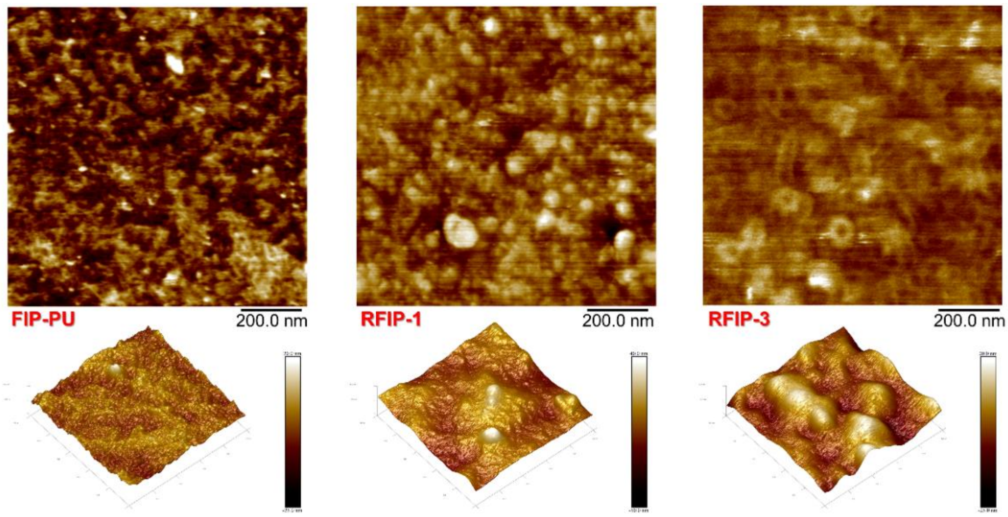

Figure S13. AFM phase images of FIP-PU, RFIP-1, and RFIP-3.

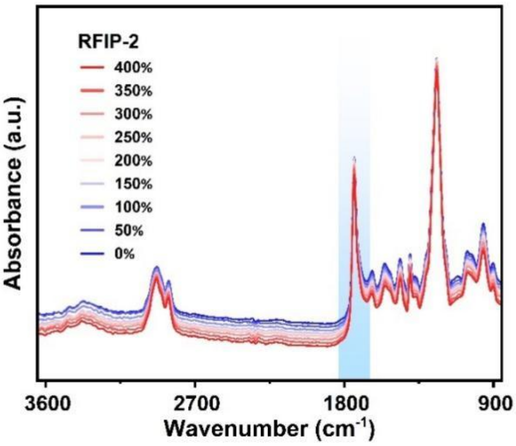

Figure S14. Stretch-related ATR-FTIR spectra of RFIP-2 in the 3600~900 cm^{-1} band.

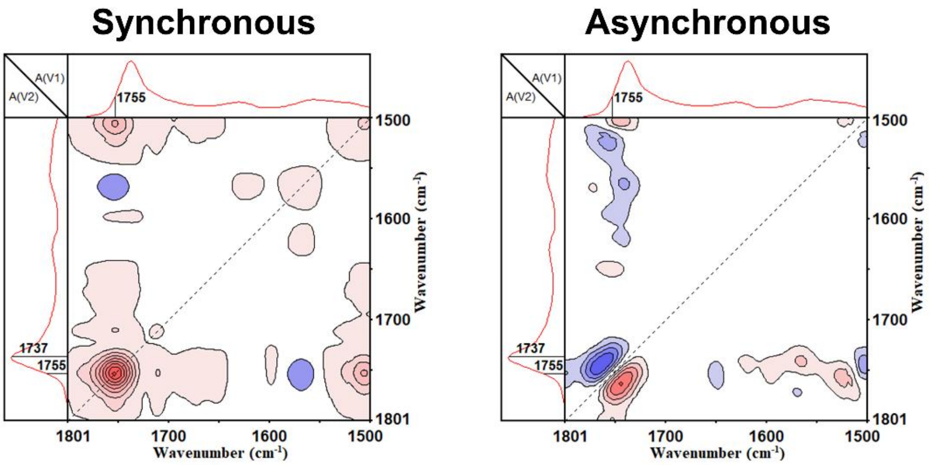

Figure S15. The synchronous and asynchronous 2D-COS spectra for the 1800~1500 cm^{-1} band of RFIP-2. Red and blue represent positive and negative, respectively.

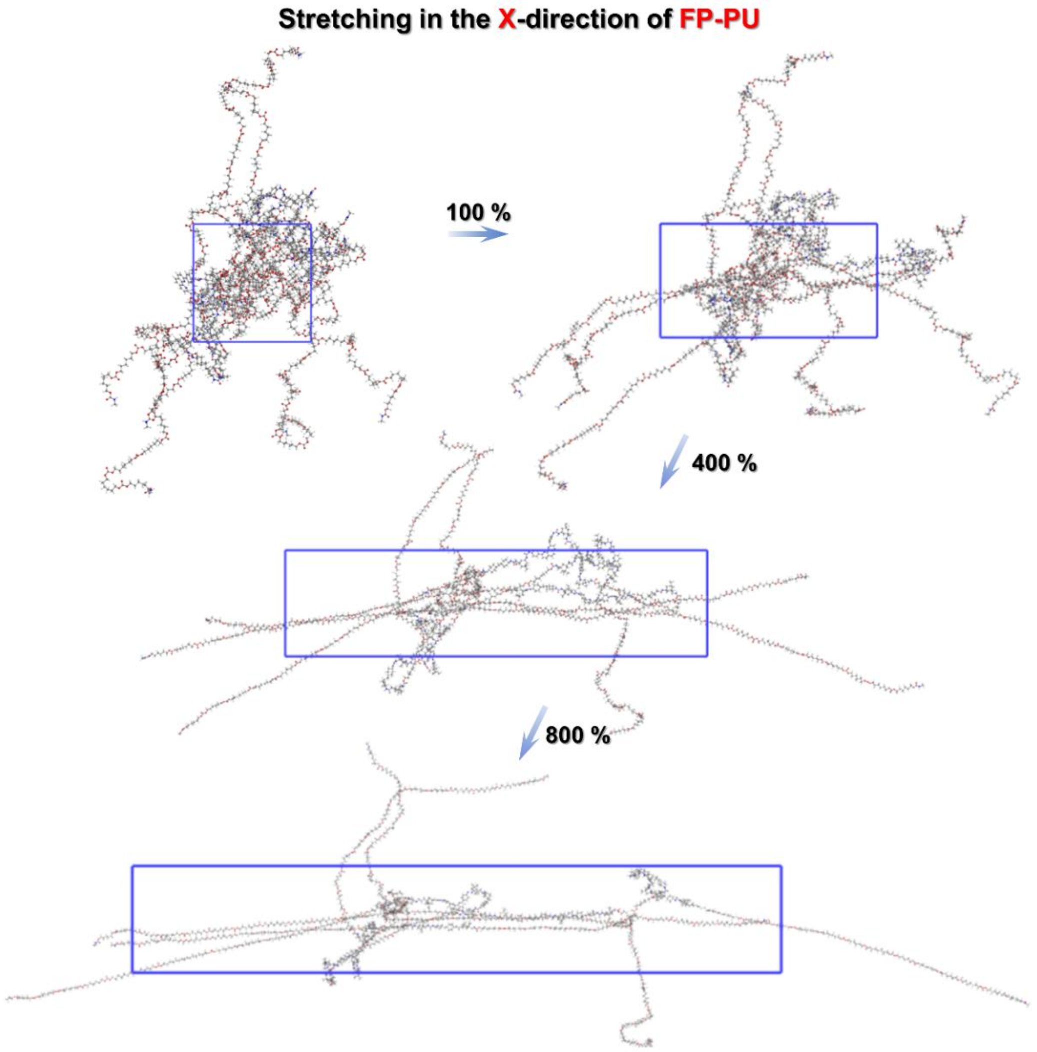

Figure S16. Snapshots showing the MD simulations of Model FP-PU at strains of 0%, 100%, 400%, and 800%.

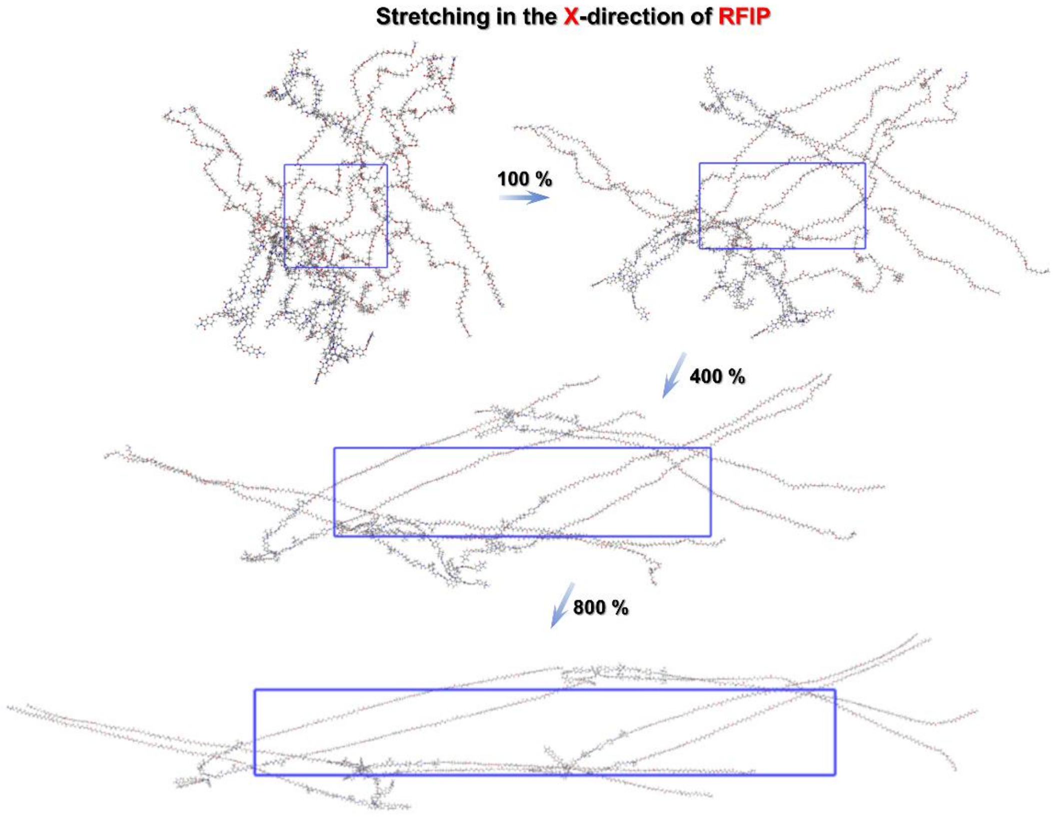

Figure S17. Snapshots showing the MD simulations of Model RFIP at strains of 0%, 100%, 400%, and 800%.

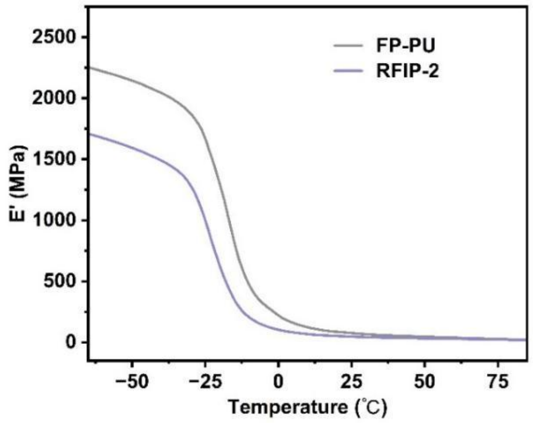

Figure S18. The Storage Modulus (E′) of RFIP-2 and FP-PU.

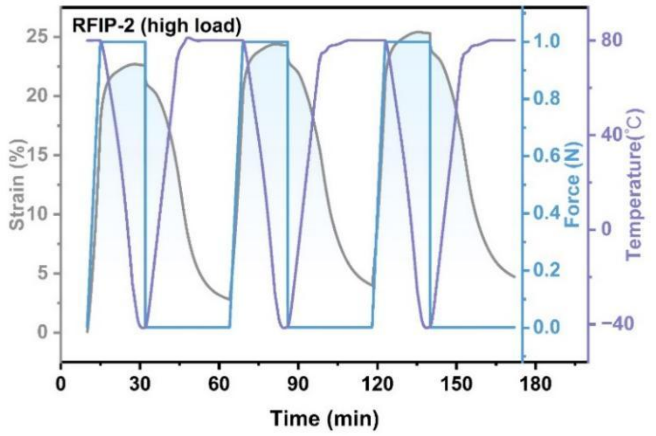

Figure S19. Shape memory cycle DMA curves of RFIP-2 under a high load of 1.0N.

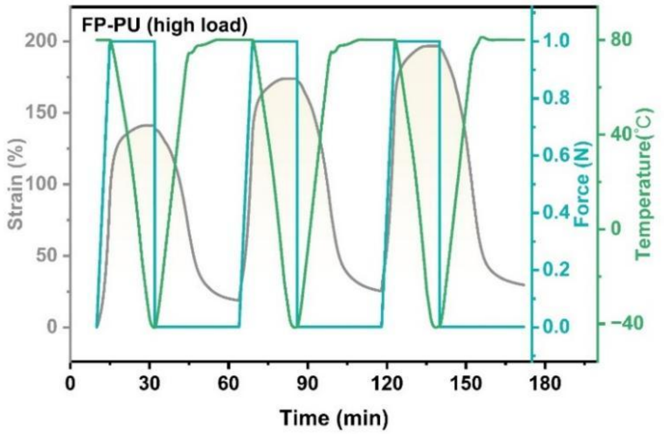

Figure S20. Shape memory cycle DMA curves of FP-PU under a high load of 1.0N.

Table S3. Summary of shape fixation rates (R_f) and shape recovery rates (R_r) for FP-PU and RFIP-2 under different loads.

Sample	Load (N)	R_f (%)	R_r (%)
FP-PU	1.0	99.1±0.6	93.3±6.6
	0.2	98.4±0.5	91.7±4.7
RFIP-2	1.0	95.9±0.4	92.9±5.3
	0.2	95.4±1.4	90.9±7.6

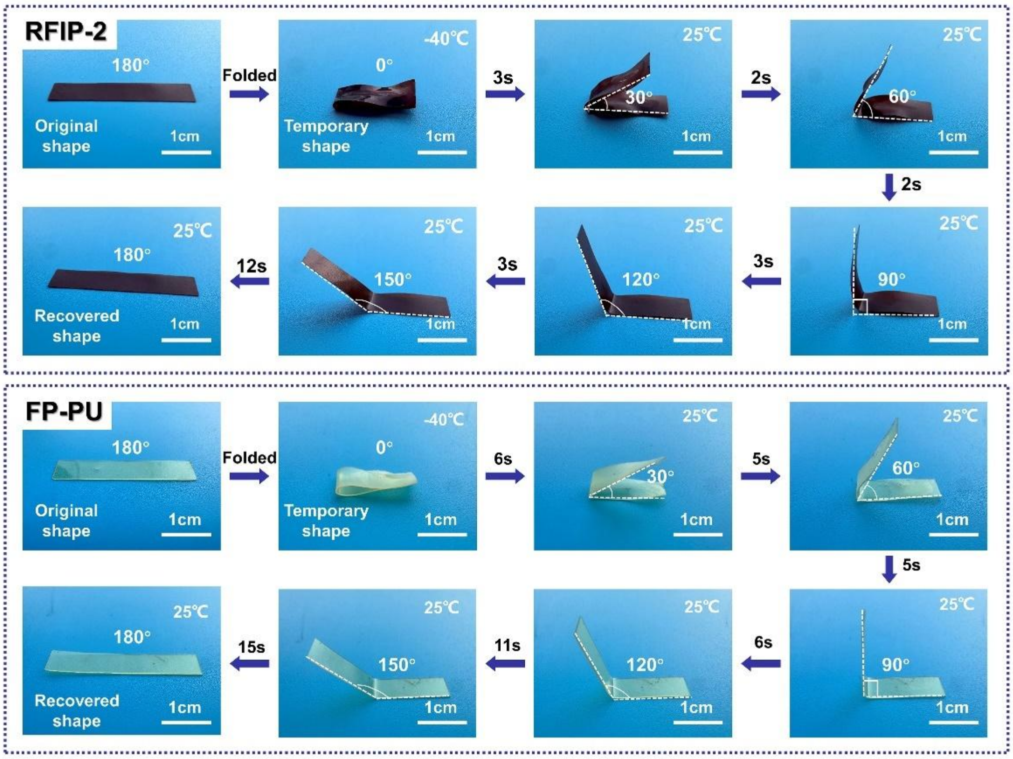

Figure S21. Photographs showing the process of shape fixation and recovery of RFIP-2 and FP-PU.

The sample was cut into a rectangular shape, then deformed into a fully folded state, and placed in liquid nitrogen at -40 °C to fix the temporary shape. Then the temporary shape was completely fixed at an angle of 0°. Lastly, the fixed samples were placed in an environment with a temperature of 25°C, and the shape memory behaviors of RFIP-2 and FP-PU were recorded respectively. (**RFIP-2:** 0°→30° takes about 3 s, 30°→60° takes about 2 s, 60°→90° takes about 2 s, 90°→120° takes about 3 s, 120°→150° takes about 3 s, and 150°→180° takes about 12 s; **FP-PU:** 0°→30° takes about 6 s, 30°→60° takes about 5 s, 60°→90° takes about 5 s, 90°→120° takes about 6 s, 120°→150° takes about 11 s, and 150°→180° takes about 15 s.)

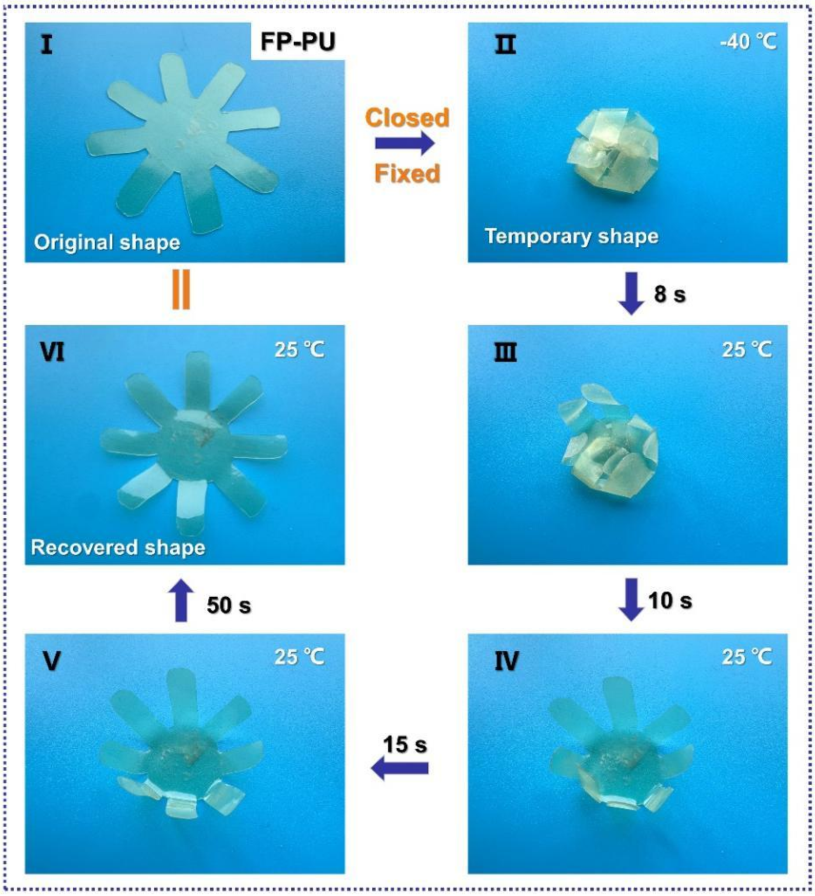

Figure S22. Photographs showing the process of shape fixation and recovery of the "sunflower" shaped FP-PU.

All petals of the “sunflower”-shaped RFIP-2 were folded inward to shape, and then placed in -40°C liquid nitrogen to fix the temporary shape. Then the fixed sample was placed in an environment with a temperature of 25°C, and the shape memory behavior of FP-PU was recorded. All closed petals gradually recovered to their original shape at 25°C, and the total time until complete recovery was about 83 s.

6. Supporting Movies

Movie S1. A rectangular RFIP-2 film can easily lift a weight of 20.0 kg

Movie S2. Cyclic tensile testing of RFIP-2 from 1st to 300th cycle.

Movie S3. Shape memory-driven forward or motion process of RFIP-2 and FP-PU.

Movie S4. Shape memory process of the rectangular RFIP-2 and FP-PU at 25 °C.

Movie S5. Shape memory process of the flower-shaped RFIP-2 and FP-PU at 25 °C.

7. Supporting References

[1] B. K. Kim, S. Y. Lee, M. Xu, *Polymer* **1996**, *37*, 5781.

[2] P. R. H. Sun, J. R. Fried, *Computational and Theoretical Polymer Science* **1998**, *8*, 229.

[3] H. Sun, *The Journal of Physical Chemistry B* **1998**, *102*, 7338.
